# Cerebrospinal Fluid Shunt Infections in Children: Do Hematologic and Cerebrospinal Fluid White Cells Examinations Correlate With the Type of Infection?

**DOI:** 10.1097/INF.0000000000003374

**Published:** 2022-03-04

**Authors:** Danilo Buonsenso, Federico Bianchi, Giancarlo Scoppettuolo, Paolo Frassanito, Luca Massimi, Massimo Caldarelli, Niccolò Salvatelli, Valentina Ferro, Piero Valentini, Gianpiero Tamburrini

**Affiliations:** From the * Department of Woman and Child Health and Public Health, Fondazione Policlinico Universitario A. Gemelli IRCCS; † Dipartimento di Scienze Biotecnologiche di Base, Cliniche Intensivologiche e Perioperatorie, Università Cattolica del Sacro Cuore; ‡ Center for Global Health Research and Studies, Università Cattolica del Sacro Cuore; § Pediatric Neurosurgery, Institute of Neurosurgery, Catholic University Medical School; ¶ Department of Infectious Diseases, Fondazione Policlinico Universitario “A. Gemelli” IRCCS; ‖ Emergency Department, Ospedale Pediatrico Bambino Gesù, Rome, Italy.

**Keywords:** cerebrospinal fluid, infections, children, antibiotics

## Abstract

**Background:**

Cerebrospinal fluid (CSF) shunt infections in children represent an increasing problem in clinical practice. However, comprehensive clinical, laboratory and microbiologic data are scarce in pediatric age.

**Methods:**

We conducted a 10-year retrospective study to (1) analyze clinical, laboratory and microbiologic parameters associated with infections in children; (2) analyze results according to the type of catheter (medicated or not), type of infection (first or relapses), type of hydrocephalus (acquired and congenital), presence or not of bacteriemia; (3) describe antibiotic susceptibilities and their evolution during the study period.

**Results:**

Eighty-seven children with shunt infection and 61 children with mechanical shunt malfunction were enrolled. Fever, vomit, leukocytosis and elevated C-reactive protein were more frequent in the infected group (*P* < 0.001), while neurologic symptoms developed more frequently in the noninfected group (10.3% vs. 27.87%; *P* = 0.006). Local signs of inflammation and abdomen distension were similarly reported in the 2 groups. Children with medicated shunts had lower cell count in the CSF (12/mm^3^) compared with those with nonmedicated shunts (380/mm^3^; *P* < 0.0001). Gram-negative bacteria were more common in the not-medicated catheters (90.91% vs. 50% of cultures; *P* = 0.04). Gram-negative bacteria were identified in 50.67% of CSF cultures, Gram-positive bacteria in 53.33% and fungi were observed in 5.33%. Sixteen children (18.4%) had also a positive blood culture. Enterococci isolation was associated with relapsed infections (37.50% vs. 15.25%; *P* = 0.05).

**Conclusions:**

Our study shows that the diagnosis and management of children with shunt infections are challenging. Prospective studies with a comprehensive approach focusing on patient, medical, microbiologic and surgical risk factors for first infection are urgently needed.

## Abstract 2

Supplemental Digital Content is available in the text.

Cerebrospinal fluid (CSF) shunting is among the most common procedures performed by pediatric neurosurgeons and represent the main surgical treatment of hydrocephalus.^1–3^ Shunt insertions for infants and children account for a considerable number of neurosurgical procedures and admission worldwide.^4^

Despite their widespread use and improvements in their management, shunt devices often malfunction due to complications such as obstruction, breakage, migration and infection.^2^ Shunt-related infections represent a relatively common cause of shunt malfunction in children,^5^ associated with both potential clinical sequelae in children^6,7^ and high costs for national health systems.^8–11^

Current evidence suggests that children with shunt infections may present with a wide range of clinical presentations, ranging from signs and symptoms of systemic infection, to manifestations of raised intracranial pressure, to even subtle presentations without fever.^12^ Similarly, blood tests may not be abnormal. Available studies did not attempt to define if specific subgroups of patients (eg, children with acquired vs. congenital hydrocephalus), nor have predictive models of infection been developed. In addition, detailed microbiologic studies of children with shunt infections are scarce.^12^

Due to current gaps of available literature, we performed this retrospective study in a regional referral University Hospital for Pediatric Neurosurgery aiming to provide a comprehensive clinical and microbiologic analyses of children with ventricular shunt infections.

## MATERIALS AND METHODS

### Study and Population Characteristics

The study was conducted retrospectively examining a cohort of children with shunts younger than 18 years of age. They were divided into 2 groups: patients with shunt infection and not infected patients. The study period was of 10 years, recruiting patients who underwent hospitalization from 2009 to 2019. The study was approved by the Institution Review Board of our Institute.

Inclusion criteria were age < 18 years; children underwent ventricular shunt placement; microbiologic data about the infection (agent, susceptibility); clinical, laboratory and outcome data were available. In our institution, we investigate for a suspected shunt infection all children with shunt and assessed for fever, new onset neurologic symptoms including vomiting and headache. For those children that had more than 1 shunt infection, we performed a subanalysis considering the second episode as a “relapsed” case. Children who underwent ventricular shunt placement not complicated by infection, hospitalized for the fever or new onset neurologic symptoms including vomiting and headache, for which clinical, laboratory and outcome data were available have been included as a control group.

### Definition of Ventricular Shunt Infections

A CSF ventricular shunt infection was defined according to the Hydrocephalus Clinical Research Network consensus definition^12^:

- microbiologic determination of bacteria present in a culture or Gram stain of CSF, wound swab and/or pseudocyst fluid;
- shunt erosion (visible hardware);
- abdominal pseudocyst (without positive culture);
- for children with ventriculoatrial shunts, presence of bacteria in a blood culture.

### Anamnestic Data

For each patient, we evaluated clinical history and anamnestic data including the age of patients, gender, nationality, any underlying disease, date of neurosurgical shunt placement and length of hospitalization as well as the etiology of hydrocephalus. Prematurity was always investigated. The type of device including whether it was premedicated or not was evaluated. If a device was removed, the date of and reason for shunt removal was always registered and, in patients with shunt infection, the date of infection detection.

### Clinical and Laboratory Features

Most common signs and symptoms in shunt infections and shunt malfunctions including fever, vomiting and neurologic symptoms, such as headache, irritability and drowsiness as well as local signs including hyperemia of the shunt tract and abdomen distension, and also respiratory distress, cyanosis and septic shock for looked for. Clinical records were analyzed to find laboratory data of all patients.

### Treatment Evaluation

All treatments were examined on clinical records and on consultancies done by infectious disease specialists. We examined empiric and targeted therapy, including length of systemic antibiotic therapy and need of intrathecal therapy.

In our Institution, all children undergoing ventricular-shunt placement received an intraoperative shot of cefazoline (30 mg/kg) followed by cefazoline 30 mg/kg/die for the following 3 days if they received an internal shunt. In case a medicated shunt is included, the postsurgery prophylaxis is not performed.

### Primary Objective

To analyze clinical, laboratory and microbiologic parameters associated with infections, in a cohort of shunt carriers’ children compared with a control group of children with shunts without infection.

### Secondary Objectives

- to analyze significant association between principal pathogens isolated in CSF and clinical and laboratory data
- to analyze microbiologic, clinical and laboratory features of children with ventricular shunts infections and according to the type of catheter (medicated or not), type of infection (first or relapses), type of hydrocephalus (acquired and congenital), presence of not of bacteremia
- to describe antibiotic susceptibilities and their evolution during the study period

### Statistical Analyses

A statistical analysis was performed using the software STATA/IC 14.2 version 2017 (StataCorp LLC). We provided a description of the set of data using proportions and percentage for categorical variables, while continuous variables were described by measures of central tendency (mean or median, depending whether the distribution was normal or not), and measures of dispersion (SD; interquartile range).

Mainly, we compared patients having infected with not infected shunt device through χ^2^ test or Fisher exact test for categorical variables or Student *t* test or Mann-Whitney *U* test for continuous variables. We also performed a multivariable logistic regression analysis exploring the potential predictors associated with patients presenting an infection of the shunt device.

We considered a 2-tailed *P* value of less than 0.05 to be significant.

## RESULTS

### Study Population

During the study period, in our institution 930 children underwent neurosurgical interventions for ventricular shunt placement. Of them, 87 children (9.3%) were admitted for a shunt infection, 70 (70.4%) had a primary infection and 17 (19.6%) a recurrent infection. At time of the infection, median patient age was 3 years. Sixty patients (69%) were male. Hydrocephalus was acquired in 38 children (43.68%) and congenital in the remaining 49 of them.

As a control group, we analyzed the data of 61 children with mechanical shunt malfunction (37 males, 60.6%, median age of 5 years), for which all data including outcome were available. Within noninfected cases, 43 (70.49%) presented congenital hydrocephalus.

Fever was present in 60 patients with shunt infection (69%), while only 2 children (2.3%) presented vomit episodes. On the other hand, vomit episodes were present in 26 children (42.62%) of the mechanical shunt malfunction group. Differences between the groups regarding fever and vomiting were statistically significant (*P* < 0.001). Conversely, local signs of inflammation and abdomen distension were similarly reported in the 2 groups, while neurologic symptoms developed more frequently in the noninfected group (10.3% vs. 27.87%; *P* = 0.006).

Leukocyte and neutrophil counts and C-reactive protein (CRP) values were significantly higher in the group of infected shunts (*P* < 0.001).

CSF analysis revealed significantly higher median values of white blood cell in CSF in the infected groups (*P* = 0.004), while no statistically significant differences were found in glucose and protein levels.

Further clinical and laboratory details are reported in Table 1.

**TABLE 1.** Differences of Baseline, Clinical and Laboratory Characteristics Between Children With Infected and Not Infected Shunt Device

Variables	Infected Shunt, N = 87 (%)	Not Infected Shunt, N = 61 (%)	*P*
Age (mo), median (IQR)	3 (8)	5 (7)	>0.05
Gender			>0.05
Male	60 (69)	37 (60.6)	
Ethnic group			>0.05
Italian	73 (83.9)	50 (82)	
Other	14 (16.1)	11 (18)	
Etiology hydrocephalus			>0.05
Acquired	38 (43.7)	18 (29.5)	
Congenital	49 (56.3)	43 (70.5)	
Prematurity at birth	21 (21.8)	23 (37.7)	**0.035**
Fever	60 (69)	0	**<0.001**
Vomit	2 (2.3)	26 (42.6)	**<0.001**
Local signs (hyperemia of the shunt tract, abdomen distension)	24 (27.9)	13 (21.3)	>0.05
Neurologic symptoms (headache, irritability, drowsiness)	9 (10.3)	17 (27.9)	**0.006**
Respiratory distress	1 (1.1)	7 (11.5)	**0.009**
White blood cell count/µL, median (IQR)	13,060 (10,180–17,640)	9425 (7150–12,065)	**0.0006**
Neutrophil count/µL, median (IQR)	8630 (5750–12,410)	5370 (2745–6790)	**<0.001**
C-reactive protein (mg/L), median (IQR)	27.2 (16–95.4)	0.95 (0.5–7.505)	**0.0001**
White blood cell count/ µL, median (IQR)	112 (3–499)	1 (1–1)	**0.004**
Glucose level in CSF (mg/dL), mean ± SD	41.69 **±** 23.24	47.8 ± 22.16	>0.05
Protein level in CSF (mg/dL), median (IQR)	69 (23–210.5)	55 (37–86)	>0.05
CSF culture			
Gram–	38 (50.67)		
Gram+	40 (53.33)		
Fungi	4 (5.33)		
Pseudomonas (CSF)	8 (10.67)		
Staphylococcus (CSF)	25 (33)		
Enterococcus (CSF)	15 (20)		
Enterobacter	9 (12)		
Klebsiella	9 (12)		
*Campylobacter coli*	11 (14.67)		
Citrobacter	3 (4)		
Serratia	2 (2.67)		
Morganella	3 (4)		
Stenotrophomonas	3 (4)		
Acinetobacter	3 (4)		
Comamonas	2 (2.67)		
Propionibacterium	1 (1.33)		

Statistically significant values are indicated in bold.

Table (Supplemental Digital Content 1, http://links.lww.com/INF/E563) provides a detailed subanalysis of children with acquired (n = 56) and congenital (n = 92) hydrocephalus, in terms of rates of infection, symptoms and laboratory results.

### First Versus Relapsed Shunt Infection

We made a subanalysis of children with a first shunt infection and those with a relapsed shunt infection (children that had already replaced their device because of a previous infection). Within 87 total cases of infection, 17 children had a relapsed one. In general, our analyses showed that children with a first or relapsed infection had similar epidemiologic, clinical and laboratory results, including need of intrathecal therapy and length of systemic antibiotic treatment. Conversely, a significant higher rate of Enterococci isolation was found in the relapsed infection compared with first infection group (37.50% vs. 15.25%; *P* = 0.05). Further details are reported in Table 2.

**TABLE 2.** Differences Between Children With a First Shunt Infection Compared With Those With Relapsed Infection

Characteristics	Original Shunt Device, N = 70	Relapsed Shunt Device, N = 17	*P*
Etiology hydrocephalus	42 (60)	10 (58.82)	>0.05
Acquired	28 (40)	7 (41.18)	
Congenital			
Need of shunt removal	55 (78.57)	15 (88.24)	>0.05
Need of intrathecal therapy	7 (10)	3 (17.65)	>0.05
Length of antibiotic systemic treatment (d), median (IQR)	30 (20–40)	14 (10–27)	>0.05
Length of hospitalization (d), median (IQR)	31 (22–41)	12 (5–25)	>0.05
CSF culture			>0.05
Gram–	30 (50.85)	8 (50)	>0.05
Gram+	29 (49.15)	11 (68.75)	>0.05
Fungi	4 (6.78)	0	>0.05
Pseudomonas	6 (10.17)	2 (12.50)	>0.05
Staphylococcus	19 (32.20)	6 (37.50)	>0.05
Enterococcus	**9 (15.25**)	**6 (37.50**)	**0.05**
Enterobacter	7 (11.86)	2 (12.50)	>0.05
Klebsiella	7 (11.86)	2 (12.50)	>0.05
*Campylobacter coli*	9 (15.25)	2 (12.50)	>0.05
Citrobacter	3 (5.08)	0	>0.05
Serratia	3 (5.08)	0	>0.05
Morganella	1 (1.69)	2 (12.50)	>0.05
Stenotrophomonas	3 (5.08)	0	>0.05
Acinetobacter	1 (1.69)	2 (12.50)	>0.05
Comamonas	1 (1.69)	1 (6.25)	>0.05
Propionibacterium	0	1 (6.25)	>0.05

Statistically significant values are indicated in bold.

### Medicated Versus Not-medicated Devices in Infected Children

We performed a subanalysis of children with a shunt infection according to the type of shunt, medicated or not-medicated. In our institute, the only medicated device which is used is a 15% clindamycin and 0.054% rifampicin system ventricular catheter (Bactiseal; Integra LifeSciences) when external ventricular shunts are needed. Therefore, to perform this subanalysis, we only compared medicated and not-medicated external ventricular shunts. In general, clinical findings such as fever and local signs of infection were slightly less frequent in patients with medicated devices, although differences were not statistically significant. Similarly, blood cell count and neutrophil count were lower in the medicated group, while median CRP values did not differ.

Children in the medicated group had lower cell count in the CSF compared with the not-medicated group (12/mm^3^ vs. 380/mm^3^; *P* < 0.0001), while glucose and protein levels did not differ significantly. Also, Gram-negative bacteria were more common in the not-medicated catheters (90.91% vs. 50% of cultures; *P* = 0.04). Further details are reported in Table 3.

**TABLE 3.** Differences Between Medicated and Not-medicated Shunt Device (External Ventricular Shunts)

Characteristics	Blood Culture+, n = 16	Blood Culture–, n = 60	*P*
Age (mo), median (IQR)	4 (1–5)	3 (1–11)	>0.05
Gender			>0.05
Female	5 (31.25)	18 (30)	
Male	11 (68.75)	42 (70)	
Ethnic group			0.06
Italian	11 (68.75)	53 (88.33)	
Other	5 (31.25)	7 (11.67)	
Etiology hydrocephalus			>0.05
Acquired	8 (50)	25 (41.67)	
Congenital	8 (50)	35 (58.33)	
Prematurity at birth	3 (18.75)	13 (21.67)	>0.05
Fever	10 (62.50)	41 (68.33)	>0.05
Vomit	0	1 (1.67)	>0.05
Local signs (hyperemia of the shunt tract, abdomen distension)	5 (31.25)	17 (28.33)	>0.05
Neurologic symptoms (headache, irritability, drowsiness)	2 (12.5)	6 (10)	>0.05
Respiratory distress	0	1 (1.67)	>0.05
White blood cell count/µL median (IQR)	13,660 (7845–19,585)	1,2480 (10,130–16,950)	>0.05
Neutrophil count/µL, median (IQR)	9195 (5005–14,700)	8495 (5790–11,700)	>0.05
C-reactive protein (mg/L), median (IQR)	17.67 (4.57–42.4)	35 (16.3–101.4)	>0.05
White blood cell in CSF/mm^3^, median (IQR)	60 (0–1126)	105 (1.5–301)	>0.05
Glucose level in CSF (mg/dL), mean ± SD	42 ± 8.26	42.48 ± 3.37	>0.05
Protein level in CSF, median (IQR)	148 (36–222)	58.5 (21–160)	>0.05

### Microbiology

Gram-negative bacteria were identified in 50.67% of CSF cultures, Gram-positive bacteria in 53.33% and fungi were observed in 5.33%. Different rates of bacterial species were analyzed. The most frequent were *Staphylococci*, which were detected in 25 cultures (33%), followed by Enterococci (20%), *Escherichia coli* (14.7%), Enterobacteria and *Klebsiella pneumoniae* as well (12% each) and *Pseudomonas aeruginosa* (10.7%). Other less frequently identified species were *Morganella morganii* and *Serratia marcescens*.

Among patients with shunt infection, 16 (18.4%) had also a positive blood culture. Most frequent pathogens detected in blood cultures were *Staphylococcus epidermidis*, *E. coli* and *Candida parapsilosis*, each one was detected in 3 children (18.75%), followed by *Candida albicans*, *Staphylococcus hominis* and *P. aeruginosa*, each identified twice (12.5%). The remaining pathogens were *S. marcescens*, *Enterobacter cloacae*, *K. pneumoniae* and *Staphylococcus capitis*, identified once each.

Table (Supplemental Digital Content 2, http://links.lww.com/INF/E564) shows that children with shunt infections with or without positive blood cultures had no significant differences in main epidemiologic, clinical and laboratory parameters.

We also assessed if specific agents detected in the CSF were linked with a specific profile of clinical symptoms, in terms of frequency of fever, vomit, neurologic symptoms and local signs of infection. We only found statistically significant differences for patients with *K. pneumoniae* infections, which less commonly experienced fever (in 33% of cases, compared with 75.7% of those with other agents, *P* = 0.016).

### Treatment

The median length of antibiotic systemic treatment was 25 days. Intrathecal antibiotic therapy was used in 10 patients (11.49%). With respect to empiric therapy, combined therapy with vancomycin and meropenem was the most used combination, adopted in 19 children (16%). The same antibiotics were those most used as targeted therapy, respectively, in 10 (8.4%) and 9 ones, respectively, used in 8.4% and 7.5% of cases. Fluconazole was used in 6.7% cases. Among the others, the most used antibiotics were ceftriaxone, oxacillin and linezolid.

### Antibiotic Resistance Patterns

We analyzed antibiotic susceptibilities of the isolated bacteria and assessed how resistances changed during the study period. Resistance rates of the most frequent pathogens are shown in Table (Supplemental Digital Content 3, http://links.lww.com/INF/E565). *S. epidermidis* resistance was analyzed in 11 antibiograms. It presented high resistance rates to clindamycin (72.73%), erythromycin (81.82%), gentamicin (72.73%) levofloxacin (73.73%), oxacillin (90.91%) and rifampicin (45.45%), while it was always susceptible to daptomycin, linezolid, tigecycline, trimethoprim/sulfamethoxazole and vancomycin. *E. coli* presented sensitivity or low resistance rates to the majority of antibiotics, with the exception of amoxicillin/clavulanic acid (resistant in 62.5% cases), ampicillin (resistant in 75% cases) and trimethoprim/sulfamethoxazole (resistant in 50% cases).

We also studied the different resistance rates through the study period, both for Gram-positive bacteria and Gram-negative bacteria (details in Supplemental Digital Content 4, http://links.lww.com/INF/E566). Within Gram-positive bacteria, 100% of *S. epidermidis* were methicillin-resistant in 2013, 2015, 2016 and 2018, only 50% in 2017 was 50%. Vancomycin-resistant Enterococci were present in 2014 (25%) and 2019 (100%), while they were absent in 2014, 2015 and 2017. Ampicillin-resistant Enterococci were highly represented in 2014 and 2015.

Regarding Gram-negatives, *P. aeruginosa* resistant to more than 2 classes of antibiotics cases were identified in 2014 and 2015, not in 2017 and 2018. Cephalosporin-resistant *E. coli* cases were identified in 2014 and in 2016, while there were no cases of carbapenem-resistant *E. coli*. In 2014 100% of *K. pneumoniae* were cephalosporin-resistant, whereas in 2015, none of them.

### Analysis of Potential Predictors of Shunt Infection

We tried to find out a predictive model associated with children presenting shunt infections, using a multivariable logistic regression, in which potential predictors were analyzed. Age and gender of children did not influence the prediction of a shunt infection, neither did the etiology of hydrocephalus. Therefore, the predictive model was weak, with CRP being the only 1 parameter that could be defined as suggestive of infection (*P* = 0.03) (see Table, Supplemental Digital Content 5, http://links.lww.com/INF/E567).

## DISCUSSION

In this study, we evaluated a large cohort of children with ventricular shunt infections and compared their clinical and laboratory characteristics with children with a group of children admitted for a mechanical shunt malfunction without infection. Although children with shunt infections had more frequently fever, raised WBC and neutrophils, CRP and CSF WBCs, multivariable models did not find variables significantly able to detect those children with shunt infections, highlighting the difficulties in the diagnosis of shunt infections in children with ventricular shunts. Importantly, there were no specific clinical characteristics associated with a specific pathogen. Different patterns of antibiotic resistance were found. In particular, we found high rates of methicillin-resistant coagulase-negative Staphylococci (the most common single pathogen), with an increasing incidence in the last years. Conversely, all isolated *Staphylococcus aureus* were methicillin sensitive. Reassuring resistance patterns were found for *E. coli* and *P. aeruginosa* infections, while *K. pneumoniae* and *Acinetobacter baumannii* had higher resistance rates.

Taken all together, our study highlights the difficulties in the management of children with ventricular shunt infections from both a clinical and microbiologic point of view, particularly in children with medicated shunts which presented, in our cohort, less frequently signs and symptoms of infection.

In fact, in our study, fever was the only symptom that significantly differentiated those with infection from those without, although it is a not-specific finding and its absence did not exclude infection. A recent review also found that fever upon presentation was present in 16% to 42% of cases.^13^ Other single-centers experience found similar results. For example, Lu et al^14^ tried to analyze external ventricular drainage (EVD) of EVD-related infection predisposing factors in pediatric population postbrain tumor surgery. Patients with a preoperative ventricular-peritoneal shunt, those with longer operation time, those who received blood transfusion, those with more frequent CSF sampling and those with longer indwelling time of EVD had higher risks of infection (*P* < 0.05).^14^

Considering CSF studies, the only significantly different CSF parameter was the higher white blood cell count compared with those not infected, while other blood markers were not particularly useful. These findings are in line with a recent review, which found that CSF pleocytosis combined with fever has a sensitivity of 82% and specificity of 99%, while CSF eosinophilia, lactic acid, serum anti-*S. epidermidis* titer, procalcitonin and CRP are nonspecific and their utility is not well established.^13^ Interestingly, Skar et al^15^ analyzed CSF cytokines in patients with shunt infections. They found an interesting trend was observed with Gram-positive infections having higher levels of the anti-inflammatory cytokine interleukin (IL)-10 as well as IL-17A and vascular endothelial growth factor compared with Gram-negative infections, although these differences did not reach statistical significance. Conversely, Gram-negative infections displayed higher levels of the pro-inflammatory cytokines IL-1β, fractalkine (CX3CL1), chemokine ligand 2 and chemokine ligand 3, although again these were not significantly different. CSF from Gram-positive and Gram-negative shunt infections had similar levels of interferon gamma (INF-γ), tumor necrosis factor-α, IL-6 and IL-8.^15^

We evaluated if some clinical or laboratory parameters were associated with a specific microorganism but no significant associations were found, with the exception of *K. pneumoniae* which was less frequently associated with the presence of fever. Similarly, Simon et al^12^ found no differences in many patient and treatment characteristics were seen between infecting organism subgroup. Another interesting finding of our study is that infections with Enterococci were more frequently associated with relapsed infections, a data not previously described in other studies. Test et al^16^ only evaluated the relationship between the causative organisms of CSF shunt infection and the timing of infection. Among 103 children in whom a CSF shunt infection developed, the causative organism was Coagulase-Negative Staphylococci in 57 (55%), *S. aureus* in 19 (18%) and Gram-negative organisms in 9 (9%). The median time to infection did not differ (*P* = 0.81) for infections caused by Coagulase Negative Stafilococci (20 days), *S. aureus* (26 days) and Gram-negative organisms (23 days).^16^

In our study, medicated shunts were only used when EVDs were needed. We found a higher probability of Gram-negative bacteria in the medicated group, confirming a previous finding from Simon et al,^12^ highlighting the importance of Gram-negative coverage in this subgroup of patients and the need of more prospective data. Also, Soleman et al^17^ found that medicated external shunts might have no major advantage in the management of shunt-related ventriculitis. These findings are in line with a recent study that prospectively assessed the durability of antimicrobial activity of antibiotic-impregnated external ventricular drains. The authors showed that antimicrobial activity of antibiotic-impregnated external ventricular drains dropped quickly in vivo and antimicrobial impregnation did not prevent colonization by susceptible strains in 9% of cases.^18^ Conversely, Lemcke et al^19^ and Talibi et al^20^ found a reduction infection rate due to the use of medicated catheters. The use of antibiotic-impregnated shunt catheters also adds value on an economic perspective according to the findings of Eymann et al.^21^

In our cohort, external shunt substitution was necessary in the majority of cases. Winkler et al^22^ also reported a rate of dysfunction of antibiotic medicated external catheters, which did not significantly differ from silver-bearing catheters. Conversely, Mostafavi et al^23^ retrospectively studied a cohort of children 148 <15 years with ventricular-peritoneal shunt infection (mean [SD] age of 21.2 [30.1] months), who underwent treatment from March 2011 to March 2018 in the main referral children hospital in Isfahan, Iran. The treatment response was significantly prominent in patients who were infected with Gram-negative bacteria (82.9%), especially with *Acinetobacter* spp. (100%) and *P. aeruginosa* (100%). The response was significantly higher in patients with CSF glucose level of greater than 40 mg/dL (83% vs. 58.1%, respectively; *P* = 0.004). They concluded that using only intravenous antibiotics was sufficiently enough for treating many children with shunt infections, especially in those infected by Gram-negative organisms and CSF glucose level of greater than 40 mg/dL.^23^ However, the authors did not provide details on etiologic agents and antibiotic resistance and how these parameters influenced the clinical presentation, recurrence and need of device removal.

We analyzed antibiotic resistance of all isolated organisms. To our knowledge, there are not studies describing resistance patterns of children with ventricular shunt infections. A recent study provided a clinical microbiologic analysis without details on resistances and changes over time,^24^ showing that coagulase-negative Staphylococci are the main etiologic agents. We found high rates of methicillin-resistant coagulase-negative Staphylococci, while none of the *S. aureus* isolated were methicillin-resistant. *K. pneumoniae* showed a multi-resistance patterns, while *P. aeruginosa* and *E. coli* were sensitive to the majority of antibiotics. Unfortunately, these trends are in agreement with other reports from Italy, showing high rates of methicillin resistance^25^ and overall national trends for both Gram-negative and positive bacteria, which according to the European Centers for Disease and Control, view Italy as one of the countries most severely involved by antibiotic resistance.^26,27^

Our study has some limitations to be addressed. The retrospective nature of the study is an intrinsic limitation of our study that did not allow us to find predictive models of shunt infection. Also, we were not able to trace some useful information, such as time from placement to infection, time from beginning of therapy to CSF sterilization.

In conclusion, our study shows that the diagnosis and management of children with shunt infections are challenging, since neither clinical nor laboratory parameters alone are accurate for the diagnosis of shunt infections. Prospective studies with a comprehensive approach focusing on patient, medical, microbiologic and surgical risk factors for first infection are urgently needed.

## ACKNOWLEDGMENTS

Statistical analyses have been performed by Dr. Valentina Ferro, II, MSc in Clinical Epidemiology.

## Supplementary Material


