# Supplementary material for: Cerebrospinal Fluid Shunt Infections in Children: Do Hematologic and Cerebrospinal Fluid White Cells Examinations Correlate With the Type of Infection?
Source: Pediatr Infect Dis J. 2022 Mar 4;41(4):324–9. doi: 10.1097/INF.0000000000003374 (PMC10863656; doi:10.1097/INF.0000000000003374)
Supplement: Supplementary file 1 [file inf-41-324-s001.docx]

**Supplemental Digital Content 1.** Differences between children with acquired or congenital hydrocephalus are analyzed. Shunt infection presented respectively in 38 patients with acquired hydrocephalus (67.86%) and 49 patients with congenital hydrocephalus (53.2%), P > 0.05. Main clinical and laboratory data did not differ in these groups, suggesting they may have a similar risk of infection and similar presentations.

|  | **Acquired Hydrocephalus**  **N 56** | **Congenital Hydrocephalus**  **N 92** | ***P* value** |
| --- | --- | --- | --- |
| **Infected device** | 38 (67.8) | 49 (53.2) | 0.08 |
| **Fever** | 25 (44.6) | 35 (38) | 0.43 |
| **Vomit** | 9(16.1) | 19 (20.6) | 0.49 |
| **Local signs (hyperemia of the shunt tract, abdomen distension)** | 17 (30.3) | 20 (21.7) | 0.24 |
| **Neurologic symptoms (headache, irritability, drowsiness)** | 9(16.1) | 17 (18.5) | 0.71 |
| **Respiratory distress** | 4 (7.1) | 4 (4.3) | 0.48 |
| **White Blood Cell Count/µL**  **median (IQR)** | 11,240 (9,520) | 11,650 (7,355) | 0.72 |
| **Neutrophil count/µL, median (IQR)** | 7,210 (7,250) | 7,880 (6,565) | 0.87 |
| **C-reactive protein** (mg/L**), median (IQR)** | 19.04 40.09) | 27.9 (88.4) | 0.27 |
| **White Blood Cell** **in CSF (unita misura), median (IQR)** | 37 (174.5) | 140 (557) | 0.09 |
| **Glucose level in CSF, mean ± SD**  **unita misura)** | 36.63 ± 3.62 | 46.12 ± 3.51 | 0.08 |
| **Protein level in CSF, median (IQR)** | 93 (162) | 61 (132.5) | 0.170 |
