# Supplementary material for: Cerebrospinal Fluid Shunt Infections in Children: Do Hematologic and Cerebrospinal Fluid White Cells Examinations Correlate With the Type of Infection?
Source: Pediatr Infect Dis J. 2022 Mar 4;41(4):324–9. doi: 10.1097/INF.0000000000003374 (PMC10863656; doi:10.1097/INF.0000000000003374)
Supplement: Supplementary file 2 [file inf-41-324-s002.docx]

# **Supplemental Digital Content 2.** Differences of baseline, clinical and laboratory characteristics between children with positive and negative blood culture in children presenting infected shunt device.

| **Characteristics** | **Medicated shunt**  **N=13** | **Not-medicated shunt**  **N=11** | ***P* value** |
| --- | --- | --- | --- |
| **Age (months),**  **median (IQR)** | 3 (14) | 4 (10) | >0.05 |
| **Gender**  Female  Male | 3 (27.3)  8 (72.7) | 3 (23.)  10 (76.9) | >0.05 |
| **Ethnic group**  Italian  other | 8 (72.7)  3 (27.3) | 10 (76.92)  3 (23.08) | >0.05 |
| **Etiology hydrocephalus**  Acquired  Congenital | 5 (45.4)  4 (54.5) | 4 (30.8)  9 (69.2) | >0.05 |
| **Prematurity at birth** | 1 (9.1) | 1 (7.7) | >0.05 |
| **Fever** | 8 (72.7) | 12 (92.3) | >0.05 |
| **Vomit** | 0 | 0 |  |
| **Local signs (hyperemia of the shunt tract, abdomen distension)** | 2 (18.2) | 5 (38.4) | >0.05 |
| **Neurologic symptoms (headache, irritability, drowsiness)** | 0 | 0 | >0.05 |
| **Respiratory distress** | 0 | 0 | >0.05 |
| **White Blood Cell Count/µL**  **median (IQR)** | 13,400 (6,680) | 17,640 (8,110) | >0.05 |
| **Neutrophil count/µL, median (IQR)** | 8,585 (3,450) | 11,700 (5,320) | >0.05 |
| **C-reactive protein** (mg/L**), median (IQR)** | 19.04 (8.7) | 19.04 (47.24) | >0.05 |
| **White Blood Cell Count/µL; median (IQR)** | 12 (123) | 380 (642) | >0.05 |
| **Glucose level in CSF, mean ± SD** | 50.3 ±0.32 | 46.36 ± 0.30 | <0.001 |
| **Protein level in CSF, median (IQR)** | 29 (70) | 79 (99) | >0.05 |
| **Need of shunt removal** | 9 (81.82) | 12 (92.31) | >0.05 |
| **Need of intrathecal therapy** | 0 | 1 (1.69) | >0.05 |
| **Length of antibiotic systemic treatment (days), median (IQR)** | 40 (30) | 35 (29.5) | >0.05 |
| **Length of hospitalization (days), median (IQR)** | 30 (30) | 37 (14) | >0.05 |
| **CSF culture** |  |  |  |
| Gram - | 5 (50) | 10 (90.9) | **0.04** |
| Gram + | 7 (70) | 3 (27.3) | 0.09 |
| Fungi | 0 | 1 (9.1) | >0.05 |
| **Pseudomonas (CSF)** | 1 (10) | 2 (18.2) | >0.05 |
| **Staphylococcus (CSF)** | 5 (50) | 0 | **0.012** |
| **Enterococcus (CSF)** | 2 (20) | 3 (27.3) | >0.05 |
| **Enterobacter (CSF)** | 2 (20) | 4 (36.3) | >0.05 |
| **Klebsiella (CSF)** | 0 | 2 (18.2) | >0.05 |
| **C. Coli (CSF)** | 0 | 1 (9.1) | >0.05 |
| **Citrobacter (CSF)** | 0 | 0 |  |
| **Serratia (CSF)** | 0 | 0 |  |
| **Morganella (CSF)** | 2 (20) | 1 (9.1) | >0.05 |
| **Stenotropomonas (CSF)** | 1 (10) | 2 (18.2) | >0.05 |
| **Acinetobacter (CSF)** | 2 (20) | 1 (0.1) | >0.05 |
| **Comamonas (CSF)** | 0 | 0 |  |
| **Propionibacterium (CSF)** | 0 | 0 |  |
