# Supplementary material for: Cerebrospinal Fluid Shunt Infections in Children: Do Hematologic and Cerebrospinal Fluid White Cells Examinations Correlate With the Type of Infection?
Source: Pediatr Infect Dis J. 2022 Mar 4;41(4):324–9. doi: 10.1097/INF.0000000000003374 (PMC10863656; doi:10.1097/INF.0000000000003374)
Supplement: Supplementary file 3 [file inf-41-324-s003.docx]

**Supplemental Digital Content 3.** Antibiotic resistance patterns of main pathogens of our cohort.

| **Antibiotic resistance rates** | | | | | | | | | | |
| --- | --- | --- | --- | --- | --- | --- | --- | --- | --- | --- |
|  | **S epidermidis (11)** | **E coli (8)** | **P aeruginosa (7)** | **S aureus (6)** | **E cloacae (5)** | **E faecium (5)** | **K pneumoniae (5)** | **E faecalis (4)** | **S capitis (3)** | **A calcoaceticus-Baumannii complex (2)** |
| **Amikacine** |  | 0% | 14.3% |  | 0% |  | 40% |  |  |  |
| **Amoxicillin/clav acid** |  | 62.5% | 85.7% |  | 100% | 20% | 100% |  |  | 50% |
| **Ampicillin** |  | 75% | 85.7% |  |  | 100% | 100% |  |  | 100% |
| **Ampicillin-sulb** |  |  |  |  |  | 100% |  | 0% |  |  |
| **Cefepime** |  | 0% | 0% |  | 0% |  | 40% |  |  | 100% |
| **Cefotaxime** |  | 12.5% | 14.3% |  | 20% |  | 60% |  |  | 50% |
| **Ceftazidime** |  | 12.5% | 42.8% |  | 20% |  | 60% |  |  | 100% |
| **Cefuroxime** |  |  |  |  |  | 80% |  | 100% |  |  |
| **Ciprofloxacin** |  | 12.5% | 14.3% |  | 0% |  | 60% |  |  | 50% |
| **Colistin** |  | 0% | 0% |  | 0% |  | 0% |  |  | 0% |
| **Clindamycin** | 72.7% |  |  | 16.7% |  |  |  |  | 100% |  |
| **Daptomycin** | 0% |  |  | 0% |  |  |  |  | 0% |  |
| **Erythromycin** | 81.8% |  |  | 16.6% |  |  |  |  | 100% |  |
| **Ertapenem** |  | 0% |  |  | 0% |  |  |  |  | 100% |
| **Gentamicin** | 72.7% | 0% | 14.3% | 0% | 0% |  | 20% |  | 100% | 50% |
| **Gentamicin HR** |  |  |  |  |  | 80% |  | 25% |  |  |
| **Imipenem** |  | 0% | 14.3% |  | 0% | 100% | 40% | 0% |  | 50% |
| **Levofloxacin** | 72.7% |  |  | 0% |  |  |  |  | 66.7% |  |
| **Linezolid** | 0% |  |  | 0% |  | 0% |  | 0% | 0% |  |
| **Meropenem** |  | 0% | 14.3% |  |  |  | 40% |  |  |  |
| **Oxacillin** | 90.9% |  |  | 0% | 0% |  |  |  | 100% |  |
| **Penicillin G** |  |  |  | 50% |  |  |  |  |  |  |
| **Piperac/Tazobac** |  | 12% | 28.6% |  | 20% |  | 100% |  |  | 50% |
| **Rifampicin** | 45.4% |  |  | 0% |  |  |  |  | 66% |  |
| **Streptomycin** |  |  |  |  |  | 80% |  | 0% |  |  |
| **Teicoplanin** | 9.1% |  |  | 0% |  | 40% |  | 0% | 33% |  |
| **Tetracycline** | 27.3% |  |  | 0% |  |  |  |  | 0% |  |
| **Tigecycline** | 0% | 0% |  | 0% |  | 0% | 0% | 0% | 0% |  |
| **Trimeth/Sulfamethoxazole** | 0% | 50% | 57.1% | 0% | 0% |  | 40% |  | 0% | 0% |
| **Vancomycine** | 0,00% |  |  | 0% |  | 40% |  | 0% | 0% |  |
