# Supplementary material for: Cerebrospinal Fluid Shunt Infections in Children: Do Hematologic and Cerebrospinal Fluid White Cells Examinations Correlate With the Type of Infection?
Source: Pediatr Infect Dis J. 2022 Mar 4;41(4):324–9. doi: 10.1097/INF.0000000000003374 (PMC10863656; doi:10.1097/INF.0000000000003374)
Supplement: Supplementary file 4 [file inf-41-324-s004.docx]

**Supplemental Digital Content 4a.** Trend in resistance of main Gram-positive pathogens during the study period.

**Supplemental Digital Content 4b.** Trend in resistance of main Gram-negative pathogens during the study period.
