# Supplementary material for: Cerebrospinal Fluid Shunt Infections in Children: Do Hematologic and Cerebrospinal Fluid White Cells Examinations Correlate With the Type of Infection?
Source: Pediatr Infect Dis J. 2022 Mar 4;41(4):324–9. doi: 10.1097/INF.0000000000003374 (PMC10863656; doi:10.1097/INF.0000000000003374)
Supplement: Supplementary file 5 [file inf-41-324-s005.docx]

# **Supplemental Digital Content 5.** Multivariable logistic regression analyzing the potential predictor associated with patients presenting infected shunt device.

| **Patient characteristics** | **Odds Ratio** | **z** | ***P* value** | **95% CI** | |
| --- | --- | --- | --- | --- | --- |
|  |  |  |  | Lower | Upper |
| **Age (months)** | 0.91 | -0.90 | 0.36 | 0.74 | 1.11 |
| **Gender (Female vs Male)** | 0.49 | -0.58 | 0.56 | 0.04 | 5.34 |
| **Vomit** | 0.0052 | -2.27 | **0.023** | 0.0001 | 0.49 |
| **Neurologic symptoms** | 1.922 | 0.35 | 0.729 | 0.048 | 77.07 |
| **Prematurity** | 0.155 | -1.27 | 0.203 | 0.009 | 2.74 |
| **Hydrocephalus (acquired vs congenital)** | 1.07 | 0.06 | 0.955 | 0.112 | 10.17 |
| **White Blood Cell Count/µL** | 1.00006 | 0.33 | 0.745 | 0.99 | 1.0004 |
| **C-reactive protein** (mg/L), | 1.203 | 2.16 | **0.030** | 1.018 | 1.42 |
